# The Prokaryotic Microbiome of *Acropora digitifera* is Stable under Short-Term Artificial Light Pollution

**DOI:** 10.3390/microorganisms8101566

**Published:** 2020-10-12

**Authors:** Jake Ivan P. Baquiran, Michael Angelou L. Nada, Celine Luisa D. Campos, Sherry Lyn G. Sayco, Patrick C. Cabaitan, Yaeli Rosenberg, Inbal Ayalon, Oren Levy, Cecilia Conaco

**Affiliations:** 1 Marine Science Institute, University of the Philippines Diliman, Quezon City 1101, Philippines; jpbaquiran@up.edu.ph (J.I.P.B.); mlnada@up.edu.ph (M.A.L.N.); campos.celine18@gmail.com (C.L.D.C.); slsayco@msi.upd.edu.ph (S.L.G.S.); pcabaitan@msi.upd.edu.ph (P.C.C.); 2 Mina and Everard Goodman Faculty of Life Sciences, Bar-Ilan University, Ramat Gan 5290002, Israel; yaelirose@gmail.com (Y.R.); inbalaya@gmail.com (I.A.); oren.levy@biu.ac.il (O.L.); 3 Israel The H. Steinitz Marine Biology Laboratory, The Interuniversity Institute for Marine Sciences of Eilat, P.O. Box 469, Eilat 88103, Israel; 4 Porter School of the Environment and Earth Sciences, Faculty of Exact Sciences, Tel Aviv University, Tel Aviv 39040, Israel

**Keywords:** 16S rRNA gene, acroporid, coral-associated microbes, ecological light pollution, Endozoicomonadaceae, holobiont

## Abstract

Corals harbor a great diversity of symbiotic microorganisms that play pivotal roles in host nutrition, reproduction, and development. Changes in the ocean environment, such as increasing exposure to artificial light at night (ALAN), may alter these relationships and result in a decline in coral health. In this study, we examined the microbiome associated with gravid specimens of the reef-building coral *Acropora digitifera.* We also assessed the temporal effects of ALAN on the coral-associated microbial community using high-throughput sequencing of the 16S rRNA gene V4 hypervariable region. The *A. digitifera* microbial community was dominated by phyla Proteobacteria, Firmicutes, and Bacteroidetes. Exposure to ALAN had no large-scale effect on the coral microbiome, although taxa affiliated with Rhodobacteraceae, Caulobacteraceae, Burkholderiaceae, Lachnospiraceae, and Ruminococcaceae were significantly enriched in corals subjected to ALAN. We further noted an increase in the relative abundance of the family Endozoicomonadaceae (*Endozoicomonas*) as the spawning period approached, regardless of light treatment. These findings highlight the stability of the *A. digitifera* microbial community under short-term artificial light pollution and provide initial insights into the response of the collective holobiont to ALAN.

## 1. Introduction

Corals play vital roles in marine ecosystems. The reefs they construct protect shorelines from wave impacts and provide habitats for diverse marine organisms [1,2]. Corals are considered holobionts or metaorganisms because of their association with photosynthetic algae (Symbiodiniaceae dinoflagellates), fungi, viruses, and prokaryotic microbes (bacteria and archaea) [3]. These symbionts reside in the corals’ surface mucopolysaccharide layer (SML), tissues, gastrovascular cavity, and skeleton [4,5,6,7,8]. These communities can vary across life stages of the coral host [9,10]. Coral-associated prokaryotic microbes are taxonomically and functionally diverse [11,12]. They are key for maintaining holobiont health as they contribute to carbon cycling, sulfur cycling, phosphorus fixation, metal homeostasis, organic remediation, the production of antibiotics, and secondary metabolite production [13,14,15]. However, environmental stressors, such as thermal anomalies, ocean acidification, eutrophication, salinity changes, chemical pollution, and sedimentation, can cause changes in the coral microbiome that disrupt these functions [16,17,18,19,20]. The loss of core microbiome functions may eventually lead to disease or death of the animal host [21].

Artificial light pollution at night (ALAN), which is also known as light pollution or photo-pollution, is an emerging stressor affecting nearshore environments and coastal zones [22,23,24]. In 2010, it was estimated that 22.2% of the world’s shorelines were already exposed to nightly light pollution [25]. The extent and intensity of ALAN impact is expected to increase as more coastal areas are developed [24,26]. The persistence of artificial and bright illumination that masks or adds to natural moonlight and starlight is a growing concern for many marine organisms, particularly corals [22,25].

Corals depend on numerous environmental cues (e.g., sea temperature, day length, wind or current patterns, and lunar cycles) to coordinate physiological processes such as metabolism, calcification, and reproduction [27,28,29]. The exposure of corals to light pollution at night has been shown to have detrimental effects on these processes. One major effect of ALAN exposure is the desynchronization of gamete release in corals [30]. This is likely linked to changes in the expression of genes related to cell cycle progression, cell proliferation, survival, and growth [31]. ALAN exposure has also been shown to have deleterious effects on coral-dinoflagellate symbiosis, resulting in severe oxidative stress, reduced symbiont cell density, lower chlorophyll concentration, and decreased maximum quantum yield of photosystem II [32,33,34]. Metabolic activity of the coral animal and its microalgal and prokaryotic symbionts are tightly intertwined [35]. Hence, artificial light exposure that affects overall holobiont metabolism may also influence the activity and abundance of closely associated microbial community members, with the potential enhancement of probiotic bacteria that can help the coral adapt to new environmental conditions. However, no studies have explored the effect of ALAN on the coral microbiome. Here, we examined the microbiome associated with gravid specimens of the reef-building coral, *Acropora digitifera*, and assessed the temporal effects of ALAN on the coral-associated microbial community. We hypothesized that the microbial assemblage would display temporal variability in response to ALAN exposure.

## 2. Materials and Methods

### 2.1. Coral Collection and Ex Situ Light Pollution Experiment

This work was part of a larger study on the effect of ALAN on coral gametogenesis and spawning. Hence, collection and experimentation were based on the known timing of gamete development and spawning for acroporids in the Bolinao-Anda Reef Complex in Pangasinan, Northwestern Philippines, as described by previous studies [36,37]. Forty-five colonies of *A. digitifera* (>30 cm in diameter) were collected from the nonlit Caniogan Reef (16°17.633′ N, 120°00.873′ E) in Anda, Pangasinan, at 4 to 5-m depths and transported to the outdoor hatchery of the Bolinao Marine Laboratory of University of the Philippines. The presence of gametes in collected corals was verified through histological analysis [36,38]. Coral sampling was conducted with permission from the Philippine Department of Agriculture–Bureau of Fisheries and Aquatic Resources with Gratuitous Permit No. 0169-19. Colonies were acclimatized in 447-L, opaque, blue plastic tanks (142 cm × 85 cm × 38 cm) with flowthrough sand-filtered seawater and natural daylight and moonlight for 21 days. Tanks were maintained at a temperature of 27.47 ± 0.47 °C (mean ± SD), salinity of 30.77 ± 0.21, dissolved oxygen (DO) of 2.36 ± 1.52 mg/L, and pH of 7.95 ± 0.07. The recorded average temperature at the reef from January to March was 28.51 ± 0.78 °C. Shade nets kept the average mid-day photosynthetically active radiation (PAR) in the tanks at 437.38 ± 363.26 µmol quanta m^−2^ s^−1^, which is within range of light levels recorded on the reef (517.47 ± 276.15 µmol quanta m^−2^ s^−1^). After the acclimatization period, coral colonies were subjected to nightly exposure to cold white LED (5329 K) with a major blue peak from 420–480 nm (“cold white” treatment) or to warm white LED (2719 K) with a major red peak from 580–620 nm (“warm white” treatment). Nighttime PAR in the tanks ranged from 0.5–0.75 µmol quanta m^−2^ s^−1^, which approximates ALAN values in populated nearshore environments, as reported by other studies [32,33,34]. Controls were subjected to natural moonlight (ambient treatment) with an average PAR of 0.01 ± 0.0 µmol quanta m^−2^ s^−1^. Each treatment was represented by three replicate tanks with five coral colonies each. Light treatments were conducted every day for up to two months from 26 January to 30 March, 2019. Lights were automatically turned on at sundown and turned off at sunrise by a photocell. Black curtains were placed between the tanks to prevent light contamination between treatments. Curtains were closed at sundown and opened at sunrise. Temperature, salinity, DO, and pH were monitored every day using a YSI Pro2030 multi-parameter meter (YSI Inc., Yellow Springs, OH, USA) and Mettler Toledo SevenGo^TM^ Duo SG78 (Mettler Toledo, Zurich, Switzerland) at 0900H and 1600H (Table S1). PAR was measured using a LI-COR LI-193 light meter (Li-Cor, Inc., Lincoln, NE, USA).

### 2.2. Sampling and DNA Extraction

Only a subset of colonies that were used in this experiment were sampled for microbial analysis. Coral fragments (1 inch in length) were sampled from the central portion of three colonies after the acclimatization period immediately before the commencement of light treatments (T1; 25 January 2019). Three colonies were sampled from each treatment after one month of exposure (T2; 25 February 2019), and three more colonies were sampled from each treatment after two months of exposure immediately before spawning (T3; 30 March 2019). Coral fragments were flash-frozen in liquid nitrogen and stored at −80 °C prior to DNA extraction. Entire coral fragments were crushed using a mortar and pestle, and total DNA was extracted using a modified cetyltrimethylammonium bromide (CTAB) method [39]. DNA was resuspended in nuclease-free water to a final volume of 30 μL and stored at −20 °C. The quality of extracted DNA was checked by agarose gel electrophoresis using 1% agarose in 1X Tris/Borate/EDTA buffer at 120 V for 20 min. DNA concentration was determined using a NanoDrop 2000c spectrophotometer (Thermo Scientific, Waltham, MA, USA).

### 2.3. 16S rRNA Gene Sequencing and Analysis

Total genomic DNA was submitted to Macrogen, Inc., Seoul, South Korea for 16S rRNA gene sequencing. The prokaryotic 16S rRNA gene V4 hypervariable region was amplified from 10 ng of DNA using the Herculase II Fusion DNA Polymerase Nextera XT Index Kit V2 (Agilent Technologies, Santa Clara, CA, USA) with primers 515F (5′-GTG CCA GCM GCC GCG GTA A-3′) and 806R (5′ GGA CTA CHV GGG TWT CTA AT-3′) [40]. Sequencing was done on the Illumina MiSeq platform with 300-bp paired-end reads. Raw sequence data were deposited in the NCBI Sequence Read Archive and can be accessed under BioProject accession number PRJNA647288. Raw data are also available at Figshare (https://doi.org/10.6084/m9.figshare.12675374.v2). Microbial community analysis was conducted using the Quantitative Insights Into Microbial Ecology 2 (QIIME2) package (2020.2) ([41], https://docs.qiime2.org/2020.2/). Quality control was carried out by denoising the sequences using the DADA2 package [42] for the correction of amplicon errors following these parameters: --p-trim-left-f 19 --p-trim-left-r 20 --p-trunc-len-f 290 --p-trunc-len-r 250. SILVA version 132 ([43]; https://arb-silva.de) was used for taxonomic classification at a 97% sequence similarity cut-off. Sequences of mitochondrial or chloroplast origin and those with <10 counts in all libraries were removed from the final set of amplicon sequence variants (ASVs).

### 2.4. Data Analyses and Visualization

Alpha diversity metrics (observed ASVs, Shannon, and Inverse Simpson) were computed using ASV counts rarefied to the smallest sample size (28,061 reads). Normality of alpha diversity data was verified using the Shapiro-Wilk test. Global differences in alpha diversity values were determined using the Kruskal-Wallis test. Variation in community composition amongst treatments was visualized using principal coordinates analysis (PCoA) based on the Bray-Curtis dissimilarity matrix. Dissimilarity in microbial community composition amongst samples was determined by permutational multivariate analysis of variance (PERMANOVA) using the adonis function in the vegan package [44] with default settings (Bray-Curtis distance and 999 permutations). To account for random effects from the repeated sampling of coral colonies, we set strata = colonyID. *p*-values were adjusted using the Benjamini-Hochberg method. Differentially abundant ASVs between pairwise combinations of treatments and timepoints were identified using ALDEx2 [45,46] on center log ratio-transformed data at an effect size threshold greater than |3|. Bacterial indicator taxa, or ASVs that were significantly associated with specific timepoints or light treatments, were identified using the indicspecies package [47] based on the relative abundance of ASVs in the rarefied dataset. All statistical analyses and data visualizations were done using vegan [44], phyloseq [48], and ggplot2 [49] packages and were performed on Rstudio version 1.2.1335 ([50]; https://rstudio.com).

## 3. Results

### 3.1. Prokaryotic Microbial Community Composition of A. digitifera

The sequencing of the 16S rRNA V4 region of the *A. digitifera*-associated microbiome yielded a total of 2,544,965 reads from 21 libraries (Table S2). A total of 1,225,029 sequences passed the filtering steps. Classification using the SILVA database returned 1063 ASVs affiliated with 24 prokaryotic phyla, 39 classes, 89 orders, and 147 families. Rarefaction curves of observed ASVs approached an asymptote for all coral samples, indicating that the sequencing depth was sufficient to cover all the possible ASVs in each sample (Figure S1).

**Figure 1:**
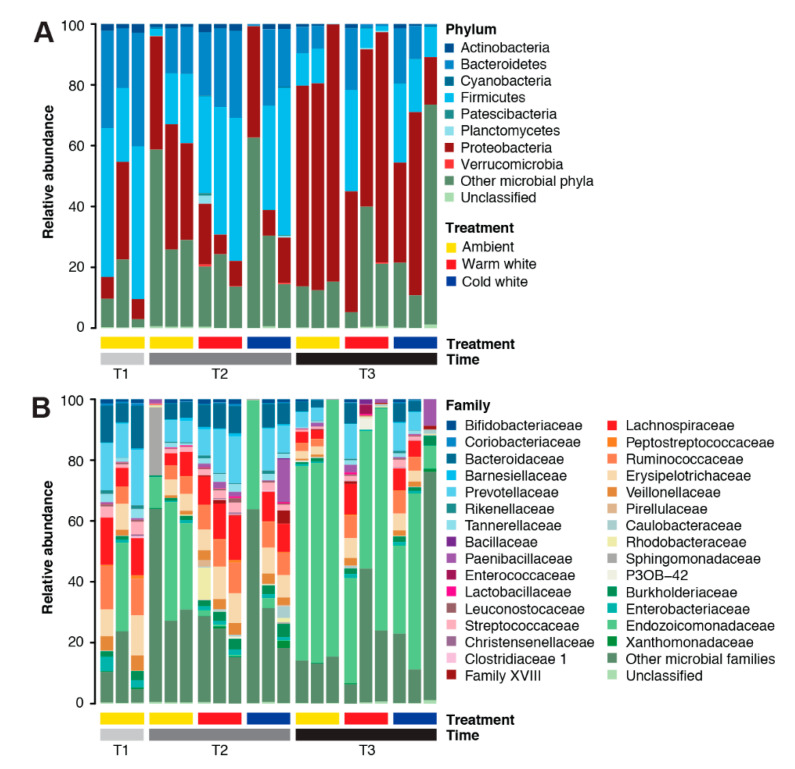
Microbial community composition of *Acropora digitifera*. Relative abundance of microbial taxa at (**A**) phylum and (**B**) family levels in corals subjected to various light treatments (ambient, warm white, and cold white) for different durations (T1, January; T2, February; and T3, March). Phyla or families representing <0.1% of the total community are represented as “Other microbial phyla” and “Other microbial families”, respectively.

The *A. digitifera* microbiome was dominated by members of the phyla Proteobacteria (35.03%), Firmicutes (25.71%), Bacteroidetes (16.44%), Actinobacteria (1.34%), Planctomycetes (0.24%), Verrucomicrobia (0.08%), and Cyanobacteria (0.07%) (Figure 1A). Dominant families in the *A. digitifera* microbiome included Endozoicomonadaceae (28.70%), Prevotellaceae (8.39%), Lachnospiraceae (6.97%), Ruminococcaceae (5.89%), Bacteroidaceae (5.64%), Erysipelotrichiaceae (5.63%), Veillonellaceae (2.03%), Burkholderiaceae (1.78%), Paenibacillaceae (1.69%), Streptococcaceae (1.62%), Tannerellaceae (1.29%), and Enterobacteriaceae (1.11%) (Figure 1B). Archaeal phyla, such as Euryarchaeota and Thaumarchaeota, were also detected but at very low counts. It should be noted that the microbial symbiont communities associated with *A. digitifera* were highly variable even for colonies subjected to the same treatments and sampled at the same timepoints.

### 3.2. Effect of Time and Light Treatment on Microbial Community Structure

**Figure 2:**
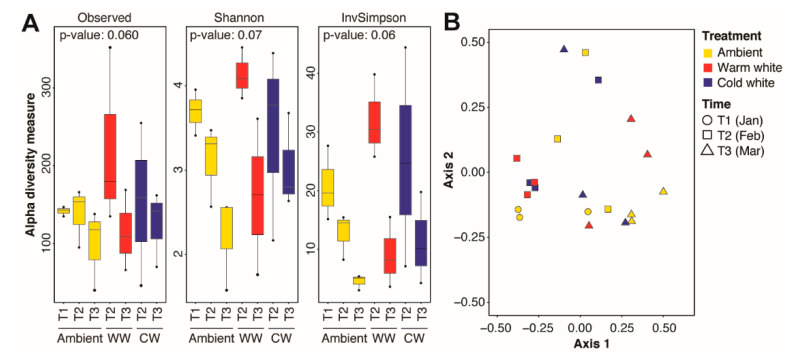
Alpha and beta diversity of the *Acropora digitifera* microbiome. (**A**) Observed amplicon sequence variants (ASVs), Shannon, and inverse Simpson indices for coral samples subjected to various light treatments (ambient moonlight, Ambient; warm white, WW; and cold white, CW) for different durations. Kruskal-Wallis test *p*-values for comparisons are shown. (**B**) Principal coordinates analysis of microbial communities in corals exposed nightly to ambient moonlight, warm white light, or cold white light for different durations.

Significant changes in microbial community diversity were observed between corals sampled at different timepoints, regardless of light treatments (Figure S2A). This was also reflected in a shift in the microbial community structure as the spawning period approached (PERMANOVA, Timepoints: *R*^2^ = 0.2299, *p* = 0.01; Table 1), with a significant difference between T2 and T3 (PERMANOVA, T2 × T3: *R*^2^ = 0.1658, *p* = 0.04; Table 1). In contrast, no significant differences in alpha diversity metrics were observed between corals subjected to different light regimes (Figure S2B) or between corals subjected to different light treatments for varying durations (Figure 2A). The microbial community structure also remained similar across light treatments (PERMANOVA, Treatments: *R*^2^ = 0.0864, *p* = 1.00; Table 1) or across treatments at different timepoints (PERMANOVA, Timepoints × Treatments: *R*^2^ = 0.0737, *p* = 0.64; Table 1; Figure 2B).

### 3.3. Microbial Taxa Significantly Associated with Time and Light Treatments

**Figure 3:**
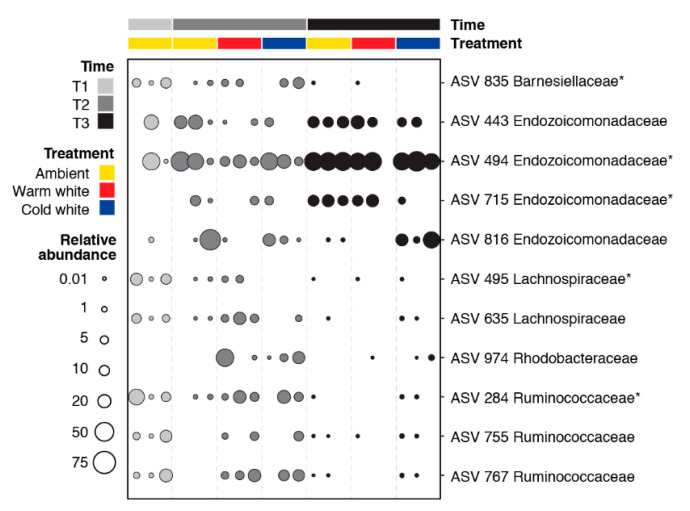
Bubble plot showing the relative abundance of 11 differentially abundant ASVs in corals exposed to different light treatments and collected at different timepoints (T1, January; T2, February; and T3, March). Differentially abundant ASVs were identified using ALDEx2 with an effect size greater than |3|. ASVs with asterisks were also identified as bacterial indicator taxa using the indicspecies package (Figure S3).

Pairwise comparisons of all time and treatment combinations using an ALDEx2 analysis with an effect size greater than |3| revealed 11 differentially abundant ASVs (Figure 3). Differentially abundant ASVs were affiliated with Barnesiellaceae (one ASV), Endozoicomonadaceae (four ASVs), Lachnospiraceae (two ASVs), Rhodobacteraceae (one ASV), and Ruminococcaceae (three ASVs). ASVs 495, 635, and 755 were relatively more abundant in the T1 samples. ASVs 284, 767, and 835 were abundant in T1 ambient and T2 light-treated samples. ASV 974 was enriched in the light-treated samples at T2. Endozoicomonadaceae ASVs (494, 443, 715, and 816) showed higher relative abundance at T2 and T3.

An indicator species analysis was also conducted as a complementary method to identify ASVs significantly associated with specific timepoints or treatments (Figure S3). This method considers the abundance and frequency of occurrence of ASVs in samples from different conditions [51]. Thirty-three ASVs, including several members of Lachnospiraceae and Ruminococcaceae, were significantly associated with corals sampled at T1, while two ASVs affiliated with Endozoicomonadaceae were associated with corals at T3. Five differentially abundant ASVs, including Barnesiellaceae (ASV 835), Endozoicomonadaceae (ASVs 494 and 715), Lachnospiraceae (ASV 495), and Ruminococcaceae (ASV 284), overlapped with ASVs identified as bacterial indicator taxa (Figure 3).

## 4. Discussion

### 4.1. The A. digitifera Microbiome Is Unaffected by Short-Term Light Pollution

The *A. digitifera* microbiome was dominated by phyla Proteobacteria, Firmicutes, and Bacteroidetes, which agrees with previous investigations on the microbiomes of adult conspecific coral species [17,52]. The microbial community structure remained stable despite nightly exposure to either warm white or cold white light. Only a few microbial taxa showed significant changes in relative abundance. However, the microbial community structure did change over time, regardless of light treatment. This shift could be related to gamete maturation as the spawning period approached or, alternatively, to changes in water quality conditions in the tanks.

The ability of the coral microbiome to tolerate stressors, such as temperature and eutrophication, has also been reported in other studies [16,17]. The compositional stability of the coral microbiome may be attributed to the physiological plasticity of each individual microbial taxon that allows it to buffer environmental change [53]. A stable microbial community ensures the continued provision of metabolites to the host and prevention of the unexpected proliferation of opportunistic or foreign bacteria that may be detrimental to the holobiont [6,54,55]. Moreover, changes in the relative abundance of some microbial taxa in the light pollution treatment is in agreement with the coral probiotic hypothesis, wherein the relative abundance of microbial species changes in a manner that allows the coral holobiont to adapt to new environmental conditions [56].

### 4.2. Microbial Taxa That Respond to ALAN

Although the overall microbial community structure remained the same in *A. digitifera* subjected to nightly light pollution, we observed an increase in the relative abundance of aerobic anoxygenic photoheterotrophic bacteria (AAPB), including members of Rhodobacteraceae and Caulobacteraceae, under the warm white and cold white light treatments, respectively. AAPB are chlorophototrophic members of the phylum Proteobacteria that contain bacteriochlorophyll *a* and use light as an alternative energy source [57,58]. Anoxygenic photosynthesis is a phototrophic process where light energy is captured and converted to ATP without the production of oxygen [59]. Members of the AAPB family can also utilize various organic and inorganic compounds, perform sulfur oxidation, carbon monoxide oxidation, and produce secondary metabolites [60]. Light directly affects and stimulates the growth rates and abundance of natural populations of marine AAPB, which explains their abundance in the corals experiencing light pollution at night [61,62]. Anoxygenic phototrophs have previously been detected in the skeletons of the scleractinian corals *Montipora monasteriata*, *Porites cylindrica,* and *Isopora palifera* [63,64].

Burkholderiaceae, Lachnospiraceae, Ruminococcaceae, and Paenibacillaceae were also enriched in *A. digitifera* fragments subjected to ALAN. It is worth noting that these microbial taxa are major taxonomic groups found in the human gut and in fecal microbiota [65,66,67,68,69], as well as in the fish gut microbiome [70,71]. The milkfish aquaculture zone and human settlements found in proximity to the Bolinao Marine Laboratory [72] are possible sources of these bacterial groups. Some members of Paenibacillaceae, although present in our coral samples, are known to be soil-associated [73] and may have been transported by coastal runoff [5].

### 4.3. Microbial Taxa Associated with Symbiodiniaceae Are Enriched under ALAN

Light pollution has been shown to affect the activity of phototrophic symbiotic dinoflagellates (Symbiodiniaceae) of corals [32,33,34]. As Symbiodiniaceae are closely associated with nitrogen-fixing bacteria (diazotrophs) [6,7,35], it is possible that ALAN exposure may also affect the abundance of these particular microorganisms. In our study, we found that members of Lachnospiraceae, Rhodobacterales, and Caulobacterales, which include known diazotrophs [74,75,76,77,78,79], were relatively more abundant in corals subjected to light at night. These bacterial groups have also been found to co-occur with dinoflagellate symbionts of corals [80,81]. It is possible that the higher abundance of symbiont-associated microbes in the corals under ALAN treatment is linked to the greater abundance and activity of the dinoflagellate symbionts.

### 4.4. Endozoicomonadaceae Abundance Increased as Spawning Period Approached

Taxa affiliated with the proteobacterial family Endozoicomonadaceae increased in abundance in *A. digitifera* coral fragments over the course of the experiment. It is likely that this increase is related to the progression of gametogenesis, as *Endozoicomonas* is found at low abundance in the microbial community of nongravid *A. digitifera* adults [17,52] or in larvae and juveniles [9] but is detected at high abundance in gravid *A. digitifera* colonies and in the egg-sperm bundles of *A. digitifera* [9] and *A. tenuis* [10]. Endozoicomonadaceae (*Endozoicomonas*) may therefore be vertically transmitted in acroporid corals [10]. *Endozoicomonas* are reported to produce quorum-sensing molecules and antimicrobial compounds [82,83], which may prevent the proliferation of non-native microbes that can harm the coral holobiont. We posit that the abundance of this bacteria in gravid corals and in their gamete bundles may serve to protect the eggs and sperm in the water column until fertilization occurs [84,85]. However, as our experimental tanks received seawater directly from the reef flat, we are unable to rule out the possibility that changes in the seawater conditions (i.e., nutrient levels and temperature) over the course of the experiment may have also contributed to the observed increase in *Endozoicomonas*. Thus, further studies are needed to explore the temporal dynamics of *Endozoicomonas* in the microbiome of *A. digitifera*.

## 5. Conclusions

One of the most understudied threats to corals is ALAN, which is an inevitable consequence of coastal development and a growing concern due to its potential negative impacts on coral reproduction. This study revealed that the overall coral microbial community structure remained stable under nightly exposure to light pollution. We did, however, observe that bacteria associated with the phototrophic symbionts of the coral, as well as those that could themselves utilize light for energy production, increased in abundance under ALAN exposure. We also noted a significant increase in the abundance of *Endozoicomonas* in the *A. digitifera* microbiome as the spawning period approached. Further studies are needed to determine whether continued exposure to ALAN, especially in combination with other stressors, affects the collective physiology and health of the coral holobiont.

## Figures and Tables

**Table 1:** Comparison of *Acropora digitifera* microbial communities at the amplicon sequence variant (ASV) level between different light treatments and timepoints. Benjamini-Hochberg-adjusted *p*-values in bold denote statistical significance at *p* < 0.05. PERMANOVA: permutational multivariate analysis of variance.

Groups	PERMANOVA	
	*R* ^2^	*p*-Value
**Global comparisons**		
Timepoints	0.2299	**0.01**
Treatments	0.0864	1.00
Timepoints × Treatments	0.0737	0.64
**Comparisons between timepoints**		
T1 × T2	0.1449	0.25
T1 × T3	0.2133	0.13
T2 × T3	0.1658	**0.04**

## Supplementary Materials

The following are available online at https://www.mdpi.com/2076-2607/8/10/1566/s1: Figure S1: Rarefaction curves for all samples based on number of ASVs identified by sequencing of the 16S rRNA V4 region. Figure S2: Alpha diversity of the *Acropora digitifera* microbiome. Figure S3: Bacterial indicator taxa for timepoints and light treatments. Table S1: Environmental conditions in experimental tanks from January to March 2019. Table S2: Information on *Acropora digitifera* colonies used in the study.
