# Supplementary material for: The Prokaryotic Microbiome of Acropora digitifera is Stable under Short-Term Artificial Light Pollution
Source: Microorganisms. 2020 Oct 12;8(10):1566. doi: 10.3390/microorganisms8101566 (PMC7601249; doi:10.3390/microorganisms8101566)
Supplement: Supplementary file 1 [file microorganisms-08-01566-s001.pdf]

Supplementary Materials

The Prokaryotic Microbiome of *Acropora digitifera* is Stable Under Short-Term Artificial Light Pollution

Jake Ivan P. Baquiran [1], Michael Angelou L. Nada [1], Celine Luisa D. Campos [1], Sherry Lyn G. Sayco [1], Patrick C. Cabaitan [1], Yaeli Rosenberg[2], Inbal Ayalon[2,3,4], Oren Levy [2] and Cecilia Conaco[1,*]

[1] Marine Science Institute, University of the Philippines Diliman, Quezon City, 1101, Philippines; jpbaquiran@up.edu.ph (J.I.P.B.); mlnada@up.edu.ph (M.A.L.N); campos.celine18@gmail.com (C.L.D.C.); slsayco@msi.upd.edu.ph (S.L.G.S.); pcabaitan@msi.upd.edu.ph (P.C.C.)

[2] Mina and Everard Goodman Faculty of Life Sciences, Bar-Ilan University, Ramat Gan, 5290002, Israel; yaelirose@gmail.com (Y.R.); inbalaya@gmail.com (I.A); oren.levy@biu.ac.il (O.L.)

[3] Israel The H. Steinitz Marine Biology Laboratory, The Interuniversity Institute for Marine Sciences of Eilat, P.O. Box 469, Eilat 88103, Israel

[4] Porter School of the Environment and Earth Sciences, Faculty of Exact Sciences, Tel Aviv University, 39040, Israel

*Correspondence: cconaco@msi.upd.edu.ph

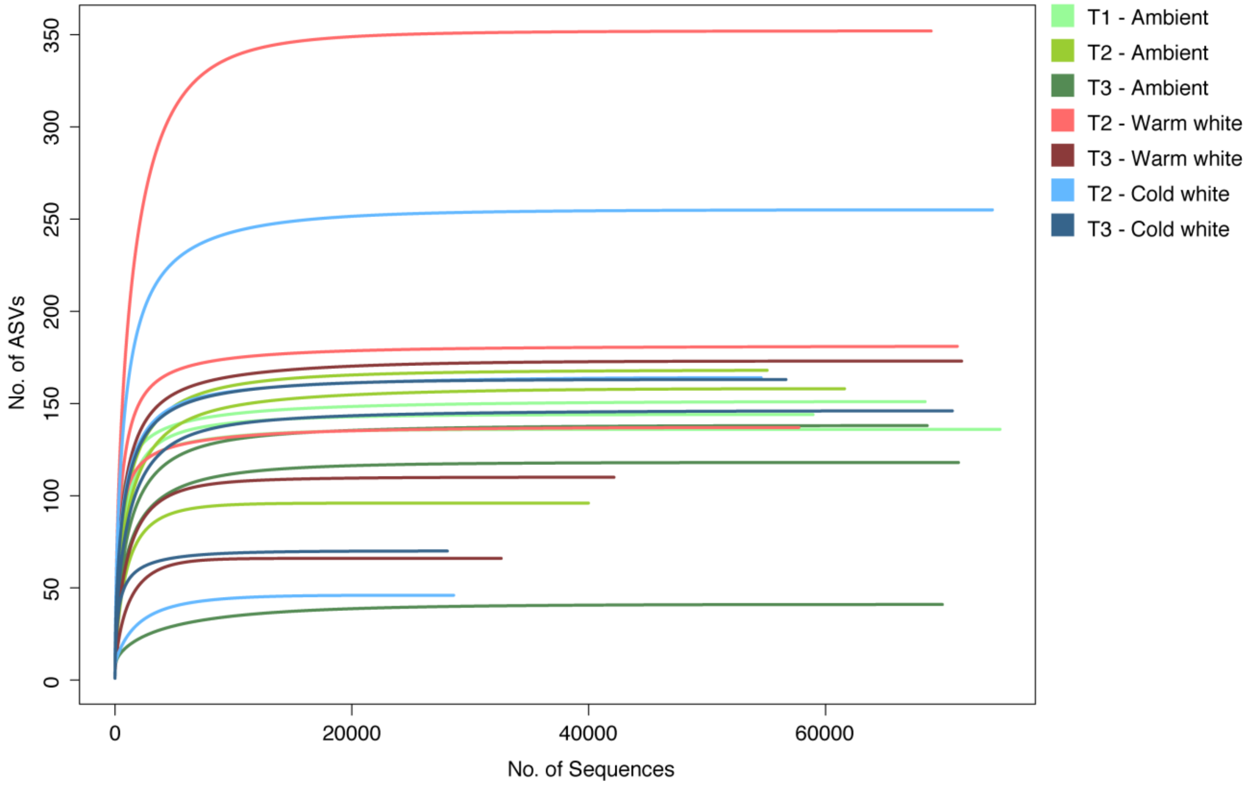

Figure S1. Rarefaction curves for all samples based on number of ASVs identified by sequencing of the 16S rRNA V4 region.

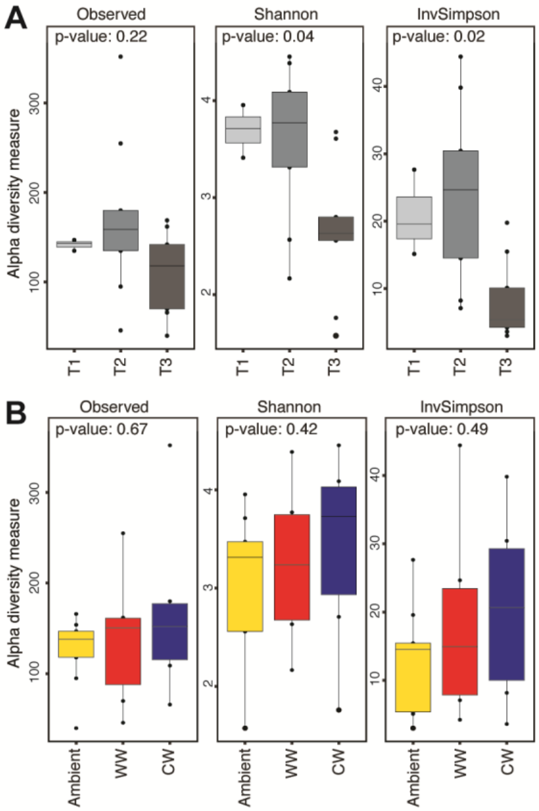

Figure S2. Alpha diversity of the *Acropora digitifera* microbiome. Observed ASVs, Shannon, and Inverse Simpson indices across (A) timepoints (T1, January; T2, February; T3, March) or (B) treatments (ambient moonlight; WW light, warm white; CW, cold white light). Kruskal-Wallis test p-values for comparisons between treatments and timepoints are shown. p-values < 0.05 indicate significant difference of the global statistics.

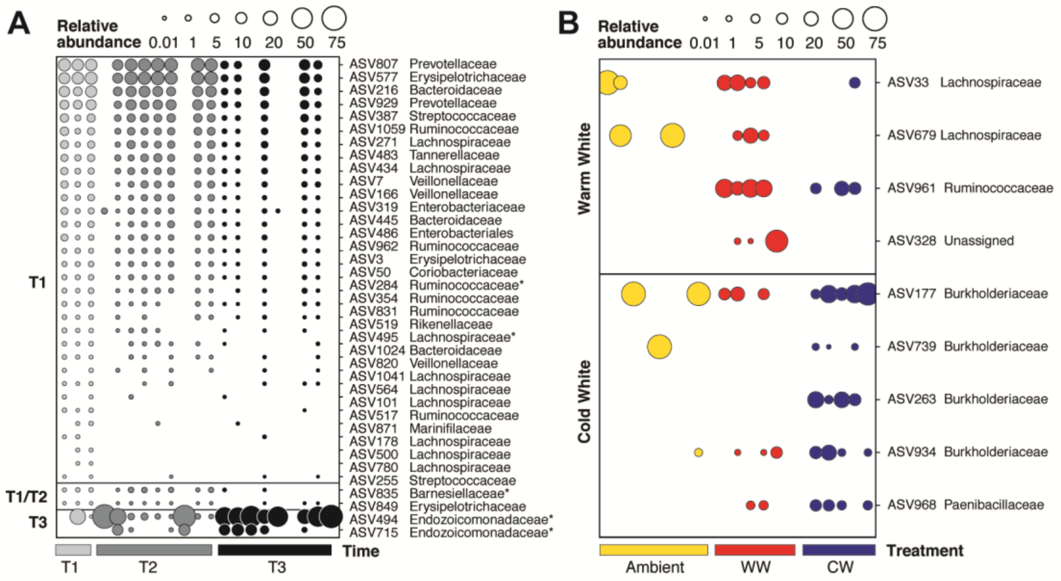

Figure S3. Bacterial indicator taxa for timepoints and light treatments. The relative abundance of ASVs significantly associated with specific (A) timepoints (T1, January; T2, February; T3, March) or (B) treatments (ambient moonlight, ambient; warm white, WW; cold white, CW), as indicated on the left side of the graphs, are shown. Bacterial indicator taxa were identified using the indicspecies algorithm (p-value <0.05). ASVs with asterisks were also identified as differentially abundant using ALDEx2 (Figure 3).

Table S1. Environmental conditions in experimental tanks from January to March 2019.

Parameter	Treatment tanks	Average (±SD)
Temperature (°C)	Ambient (T1)	26.80 ± 0.16
	Ambient (T2)	27.34 ± 0.28
	Ambient (T3)	28.79 ± 0.44
	Cold white (T1)	26.52 ± 0.45
	Cold white (T2)	27.19 ± 0.35
	Cold white (T3)	28.57 ± 0.25
	Warm white (T1)	27.03 ± 0.17
	Warm white (T2)	27.21 ± 0.33
	Warm white (T3)	28.65 ± 0.25
Salinity	Ambient	31.33 ± 0.74
	Cold white	31.48 ± 0.71
	Warm white	31.42 ± 0.73
pH	Ambient	7.90 ± 0.14
	Cold white	7.92 ± 0.13
	Warm white	7.89 ± 0.13
DO (mg/L)	Ambient	4.70 ± 2.25
	Cold white	4. 57 ± 2.04
	Warm white	4.42 ± 2.09

Table S2. Information on *Acropora digitifera* colonies used in the study.

Sample name	Colony ID	Time	Treatment
Ambient January_1	DA42	T1-January (25 January 2019; after 3 weeks acclimatization)	Ambient
Ambient January_2	DA43		
Ambient January_3	DB3		
Cold white February_1	DB3	T2-February (25 February 2019; 1 month exposure)	Cold white
Cold white February_2	DB4		
Cold white February_3	DB5		
Warm white February_1	DR22		Warm white
Warm white February_2	DR23		
Warm white February_3	DR24		
Ambient February_1	DA41		Ambient
Ambient February_2	DA43		
Ambient February_3	DA44		
Cold white March_1	DB2	T3-March (30 March 2019; 2 months exposure; spawning night)	Cold white
Cold white March_2	DB3		
Cold white March_3	DB4		
Warm white March_1	DR22		Warm white
Warm white March_2	DR23		
Warm whiteMarch_3	DR24		
Ambient March_1	DA41		Ambient
Ambient March_2	DA42		
Ambient March_3	DA43		
